# A Comparative Study on the Dissolution of *Argema mimosae* Silk Fibroin and Fabrication of Films and Nanofibers

**DOI:** 10.3390/polym13040549

**Published:** 2021-02-12

**Authors:** Hlobsile Kgomo, Somandla Ncube, Vimbai Mhuka, Temesgen Girma Kebede, Simiso Dube, Mathew M. Nindi

**Affiliations:** Department of Chemistry, The Science Campus, University of South Africa, Private Bag X6, Florida 1710, South Africa; 43369952@mylife.unis.ac.za (H.K.); ncubes@unisa.ac.za (S.N.); vimmhuka@gmail.com (V.M.); kebedtgk@unisa.ac.za (T.G.K.); nindimm@unisa.ac.za (M.M.N.)

**Keywords:** *Argema mimosae*, silk, dissolution, films, electrospinning, nanofibers

## Abstract

Limited studies have been done on silk fibroins of wild silkworm species owing to their relative insolubility in many solvents. In this study, the solubility of *Argema mimosae* wild silk fibroin in different salts (LiBr, LiCl, Ca(NO_3_)_2_, and CaCl_2_) dissolved in formic acid under varying temperatures was investigated. The dissolution conditions under which the solubility was optimum were optimized using a central composite design approach. The optimum range for solvation of the fibroin were visualized using contour plots. The influence of temperature and salt concentration were found to significantly influence the solvation of the fibroin. Following the successful dissolution of the fibroin, the regenerated silk fibroin solutions were cast to obtain water insoluble films which were used in investigating optimum electrospinning conditions. Average nanofiber diameters in the 110–141 nm range were obtained under optimum electrospinning conditions. The silk forms were characterized using the FTIR, TGA, XRD, and SEM to understand their properties. The investigations revealed that formic acid—salt solvents were effective in the solvation of the wild silk fibroin. Some of the dissolution conditions induced mild effects on the silk fibroin while others were harsh. Furthermore, processing to nanofibers resulted in the degradation of the β-sheets producing nanofibers rich in α-helices. However, post-treatment using methanol and water vapor were effective in restoring β-sheet crystallinity.

## 1. Introduction

Silk is a natural protein polymer produced by various insects including silkworms. Interest in silk fibroin (SF) from silkworms is due to its outstanding mechanical properties, high thermal stability, and other superior properties including biodegradability. As a result, silk fiber has evolved into a fascinating material that has been used mainly as sutures in the medical field over the years because of its biodegradability [1]. It has also been explored for potential application in different areas including such as tissue engineering and controlled drug delivery [2,3]. The general approach in obtaining pure SF is via a degumming process to remove sericin. This sericin is a hydrophilic protein that binds the SF fibers together to form a compact cocoon. A necessary and important step after degumming is the dissolution of the fiber to obtain a regenerated silk fibroin (RSF) solution used to fabricate various forms such as powders, gels, films, nanofibers, and membranes depending on the intended application. Generally, films are prepared by casting a RSF solution obtained from aqueous, acidic, and ionic solutions and allowing the solvent to evaporate. The electrospinning technique is an attractive and versatile technique for the fabrication of nanofibers from natural polymer materials such as silk. The technique is a simple and effective method for fabricating nanofibers with a diameter range from micro- to nanometers. Various reviews on electrospinning have highlighted advantages of this technique, notably, the production of nano-scale fibers with a large surface area to volume ratio which has been found to improve their applicability and performance in a variety of applications [4,5,6,7].

While the degumming of SF is a relatively straight-forward process, the challenge is its limited solubility in water, aqueous salt solutions, and most organic solvents. Also, most studies on the fabrication of SF materials have been done on silkworms reared for commercial purposes referred to as cultivated or domesticated silkworms. Silk fibroin from the domesticated *Bombyx mori* (mulberry silk) has been the most studied and utilized over the years. Sub-Saharan Africa is a host to various wild silk moths such as *Gonometa postica*, *Gonometa rufobrunnae*, *Argema mimosa*, and *Anaphe panda*. However, only a few studies have been carried out to explore the functionality and applicability of wild silks [8,9]. The traditional fabrication route typically involves dissolving SF in concentrated aqueous salts. Still, the dissolution of some wild SF in the aqueous salts is difficult and limited with the need for harsh conditions such as concentrations of salt as high as 10 mol dm^−3^, high temperatures, and long preparation times. The difficulty in dissolving some fibers is associated with the amino acid sequence. For instance, wild silk fibroin of *Argema mimosae* constitutes of poly-alanine (Ala) sequences and it has been suggested that poly(Ala) repeats interlock with adjacent chains as a result of hydrophobic interactions. This has been hypothesized to result in fibers with greater binding energy and thus less solubility [10,11]. On the other hand, silk fibroins with appreciable solubility in concentrated salt solutions generally constitute of poly-glycine-glycine (Gly-Gly) and poly-glycine-alanine (Gly-Ala) repeats.

In this study, silk fibroin from *Argema mimosae*, a wild silkworm species was used as the model. To our knowledge, there are no reports in literature describing silk forms derived from wild SF of the species. This may be due to its limited solubility in the traditional solvents which limits further processing. Therefore, dissolution studies were carried out in which an alternative method which involves dissolving the salt (LiBr, CaCl_2_, Ca(NO_3_)_2_, and LiCl) in formic acid (FA) rather than in water and using the salt-FA solvent to obtain a regenerated silk fibroin (RSF) solution was investigated. The optimum dissolution conditions were optimized using a central composite design approach and the range under which the dissolution was optimum was visualized using contour plots. The RSF was used for the fabrication of films and nanofibers. The degummed silk fibroin was characterized before and after processing into the films and nanofibers were investigated to assess possible processing induced effects on the silk fibroin and their effects on the silk fibroin properties.

## 2. Results and Discussion

### 2.1. Dissolution of Silk Fibroin

**Figure 1:**
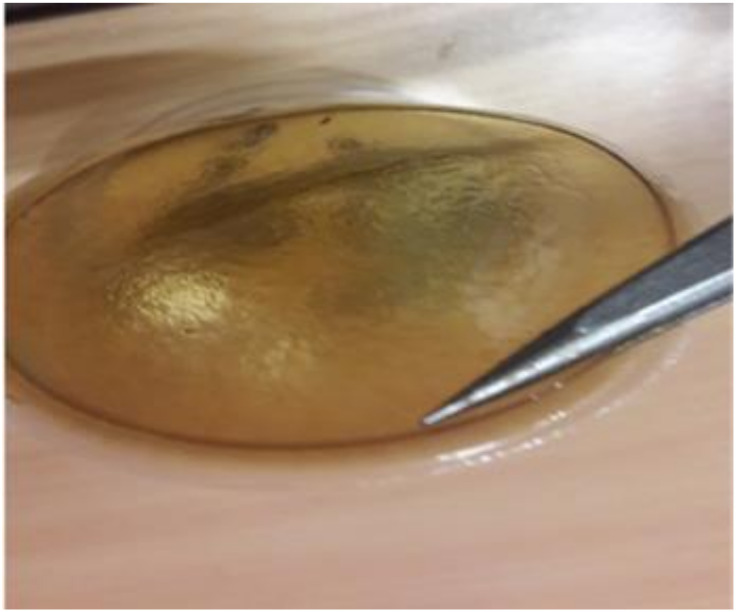
Silk fibroin film obtained from solvent systems with good solvation power.

Formic acid on its own is incapable of dissolving the fiber but only caused it to swell. The swelling of the fiber allows the salt ions to infiltrate the very stable structure of the crystalline region resulting in the disassembly of the structure first into micro-sized fibrils and further into nanosized fibrils [12]. It was observed that on dissolving the fiber, the color of the resultant RSF solutions ranged from pale yellow to amber. Generally, results were classified according to three scenarios. The first one being “no dissolution” which was characterized by a swollen fiber, but no RSF solution could be collected, therefore no films were obtained from such systems. In another scenario, there was “poor dissolution” of fibroin. In such cases, some RSF solution, usually characterized by a pale-yellow solution, could be collected for casting. However, in some cases the solution would be insufficient for film formation; as a result, no films would be obtained. Finally, solvent systems with sufficient solvation power resulted in the formation of SF films ranging from yellow to amber as shown in Figure 1.

#### 2.1.1. Effect of Salt Type

**Figure 2:**
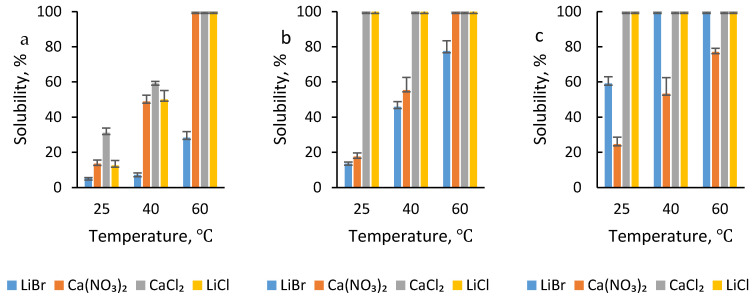
Solubility of silk fibroin at varying temperatures using salt–formic acid (FA) acid concentrations of (**a**) 1, (**b**) 3, and (**c**) 5 wt%.

The use of different salts induced different effects on the solvation of the silk fibroin as depicted in Figure 2 which shows the influence of the salt type at different temperatures using 1, 3 and 5 wt% salt concentrations. Although the solvation power of salt ions depends on both the cations and anions studies suggest that the anions play a more significant role in protein solvation as compared to the cation [13]. In this study salts containing chloride ions showed superior solvation power resulting in complete dissolution under most of the conditions investigated. This implies that Cl^−^ had a stronger ability to disrupt the hydrogen bonds of the crystalline structure. Br^−^ and NO₃^−^ are more nucleophilic and ordinarily, should give higher solvation of the fiber than Cl^−^ as was the case in work independently done by Mhuka and Addis [8,14]. In both their instances, it was observed that concentrated solutions of LiBr induced a significantly higher percentage solubility than either CaCl_2_ or LiCl salts. However, in this study, the salts were dissolved in formic acid instead of water and due to the nucleophilicity of the Br^−^ and NO₃^−^, they are highly solvated with hydrogen bonding of the solvent molecules. Thus, they are more tightly bound as compared to Cl^−^. Consequently, Cl^−^ can readily move to interact with the electrophilic centers of silk fibroin resulting in the greater and more rapid solvation of the fiber.

#### 2.1.2. Effect of Salt Concentration

The concentration of the salt has long been found to play a very important role in the solvation of fibers. High salt concentrations result in greater ionic strength and this tends to increase the solvation power of the solvent system. This proved to be true also for the dissolution of *A. mimosae* fibroin; the higher the salt concentration the higher the dissolution efficiency in most cases, as shown in Figure 2. However, that was not the case with Ca(NO_3_)_2_ that is, the higher the concentration of the salt the lower the dissolution efficiency, making 1 wt% Ca(NO_3_)_2_ the most effective concentration. The reason for this was the presence of water molecules in the dissolution system as the salt was hydrated. The higher the concentration of the salt, the more water molecules present in the dissolution system. Therefore, the solvation power was likely to be negatively affected by the water molecules because water molecules tend to compete with the electrophilic centers of silk fibroin for nucleophilic anions of salts resulting in poor solvation of the fiber [15]. As a result, poor quality films were obtained for 5 wt% salt systems with no resultant films produced for 5 wt% Ca(NO_3_)_2_ at 25 °C.

Excellent dissolution was achieved for solvent systems with 5 wt% LiCl; however, upon immersing films in water, the color of the films turned whitish with a slight layer of shiny golden brown. This might have been due to the deterioration of silk fibroin because of the sorption of water molecules as a result of the hygroscopicity of LiCl salt. It has been reported that LiCl salt is incorporated into some biopolymers in cases where water sorption is required. The salt is further used to facilitate the water adsorption capacity of fibers. In a study by Li and co-workers, it was found that the addition of LiCl enhanced the sorption of water vapor, furthermore, they observed that the water sorption improved with increasing salt concentration [16]. Similar effects are envisaged for LiCl dissolution systems, implying that at low LiCl concentrations (<5 wt%), the water sorption of the SF films was low. But as the concentration of LiCl increased, the water sorption capacity also increased resulting in high moisture content, which may have then resulted in some deterioration. A similar observation was noted for films produced using 5 wt% CaCl_2_ at 40 and 60 °C. The films appeared white with a light shiny golden-brown layer similar to those of LiCl. The difference was that upon washing with water and subsequent drying, CaCl_2_ films were dry and hard as compared to LiCl films which were soggy. Interestingly, the films produced using 5 wt% CaCl_2_ at an ambient temperature maintained the amber color. The chloride ions appeared to have a harsh effect on fiber, leading to the whitish appearance of the SF films which could be due to possible degradation. Various studies have also reported CaCl_2_ to have superior dissolution properties when compared to other salts such as LiBr; however, this was at the expense of the integrity of the SF as degradation of the fiber during processing was observed [17].

#### 2.1.3. Effect of Temperature

The lowest percentage dissolution for all solvent systems was observed for dissolution carried out under ambient temperature. An increase in temperature increased the solvation power of the systems, which in turn increased the rate and percentage of fiber dissolved.

#### 2.1.4. The Interactive Effects of Variables on Dissolution

**Figure 3:**
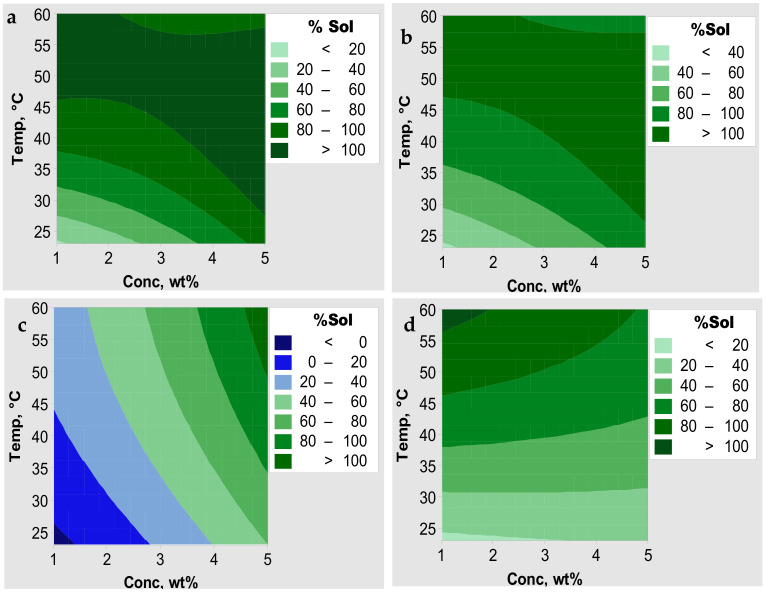
Contour plots for solvation of SF in (**a**) LiCl, (**b**) CaCl_2_, (**c**) LiBr, and (**d**) Ca(NO_3_)_2_ salt–FA solvent systems.

The solubility of the fibroin in response to the effects of temperature and salt concentration as a function of salt type was further investigated using central composite designs. The results are depicted using contour plots (Figure 3) used to investigate the range of conditions under which solvation of fibroin was optimum. It can be observed from the contour plots that salt concentration and temperature brought about different responses in the solvation of the silk fibroin. The contour plot for LiCl (Figure 3a) shows a rising ridge until a maximum is obtained in which there was 100% dissolution of the fibroin. This region occurs throughout the salt concentration range depending on the processing temperature. It was observed that between 1 and 3 wt% the lower temperature of the maxima remains almost constant at about 45 °C. Above 3 wt% the lower temperature drops linearly to about 30 °C at 5 wt% salt concentration. This optimum range was achieved when solvation of the fibroin was carried out using low salt concentration with processing temperatures above 45 °C and as the concentration of salt increased optimum dissolution was achieved even at low processing temperatures below 25 °C. A similar trend was observed for dissolution using CaCl_2_ (Figure 3b). The contour plot for LiBr (Figure 3c) shows a pattern in response to an increase in both the concentration and temperature. The initial response observed at the bottom left of the graph represents a region in which the fibroin showed no dissolution. A slight improvement of ≤20% solvation is observed with increasing salt concentration and temperature. The plot also shows that better solubility of the fiber (≥80%) was obtained when salt concentrations were greater than 4% with temperatures higher than 50 °C. The Ca(NO_3_)_2_ plot (Figure 3d), on the other hand, shows the opposite results to those of LiBr. That is, a negative rising ridge pattern was observed in which the strongest solvation power occurred between 1 and 2 wt% at temperatures above 55 °C. The contour plots indicate the influence of salt in the solvation of the fibroin in with Cl^−^ based salt systems offering optimum conditions over a wider range of conditions.

**Figure 4:**
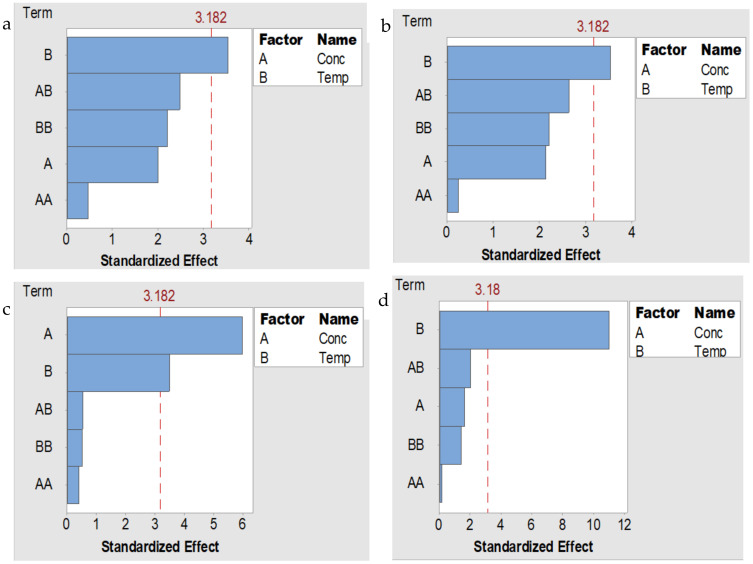
Pareto chart of standardized effects of concentration and temperature on solvation of silk fibroin in (**a**) LiCl, (**b**) CaCl_2_, (**c**) LiBr, and (**d**) Ca(NO_3_)_2_-FA solvent systems.

The Pareto analysis was used to investigate the magnitude of the effects of the different variables (salt concentration and temperature) on the solvation of the fibroin. Additionally, the variables that caused a statistically significant effect on the solvation of the fibroin at 95% confidence level are indicated as shown in Figure 4. Generally, the results show that both salt concentration and the temperature had some effect on the solvation of fibroin. For the solvation of SF using LiCl, CaCl_2_, and Ca(NO_3_)_2_ salts, the temperature induced a statistically significant effect. While with the LiBr systems, the independent effects of concentration and temperature were statistically with concentration being the more dominant variable.

### 2.2. The Effects of Electrospinning Parameters on the Morphology of Nanofibers

**Figure 5:**
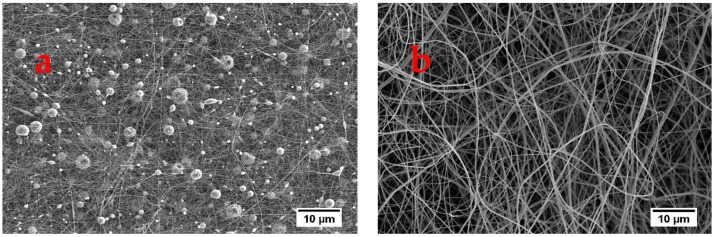
SEM images of electrospun nanofibers of Ca(NO_3_)_2_ films using polymer concentrations of (**a**) 8 and (**b**) 10 wt%.

In this study, the films produced from low salt concentrations were generally preferred. This choice was based on processing induced effects on the properties (Section 2.3) and structure (Section 2.3) of silk fibroin SF, as well as the range under-which dissolution was optimum. Therefore, electrospinning was carried out using films fabricated from 1 wt% Ca(NO_3_)_2_, CaCl_2_, and 1 wt% LiCl processed at 60 °C. For LiBr on the other hand, films produced from 5 wt% LiBr at 60 °C were used for the preparation of electrospinning solution since the best dissolution conditions were achieved under these conditions. The effect of polymer solution concentration on the morphology of the electrospun nanofibers was investigated by increasing the concentration from 6 to 17 wt%. The SEM images showing nanofibers spun at different concentrations given in Figure 5. At polymer concentrations up to 8 wt% beaded nanofibers were produced. Upon increasing the concentration to 10 wt% beadless nanofibers were collected. An increase in the concentration also increases the viscosity of the polymer solution. This in turn increases the number of polymer chains per unit volume which causes chain entanglements among the polymer chains, thus the jet at the tip of the needle can sufficiently stretch and elongate to produce beadless nanofibers [18,19]. At low concentrations, the elasticity and stretching capability of the polymer solution are too low, thus it cannot elongate sufficiently resulting in the formation of beaded fibers. Therefore, 10 wt% was used for the LiBr, Ca(NO_3_)_2_, and CaCl_2_ films, while 13 wt% was the most suitable for LiCl.

**Figure 6:**
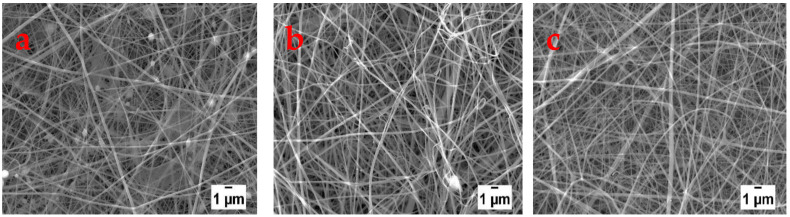
SEM images showing nanofibers produced from LiBr films at flow rates of (**a**) 4, (**b**) 6, and (**c**) 8 uL min^−1^.

Figure 6 shows SEM images depicting the influence of the flow rate on the morphology of the electrospun nanofibers. When the flow rate was 4 µL min^−1^ highly beaded fibers were collected while at flow rates of 6 to 8 µL min^−1^ the nanofibers were beadless. Further increase of the flow rate to 10 µL min^−1^ and above resulted in the collection of beaded morphologies. Other researchers have also reported that high flow rates result in nanofibers with defects, as a result, lower flow rates are preferred [7,19]. However, it should not be too low such that there is insufficient polymer solution feeding to the tip of the needle as this may also result in fibers with defects, as was the case at the flow rate of 4 µL min^−1^ in this study. At flow rates of 6 and 8 µL/min, average diameters of 110 ± 41 and 175 ± 85 nm, respectively, were obtained for nanofibers. Therefore, flow rate of 6 µL/min was chosen to be the optimum.

**Figure 7:**
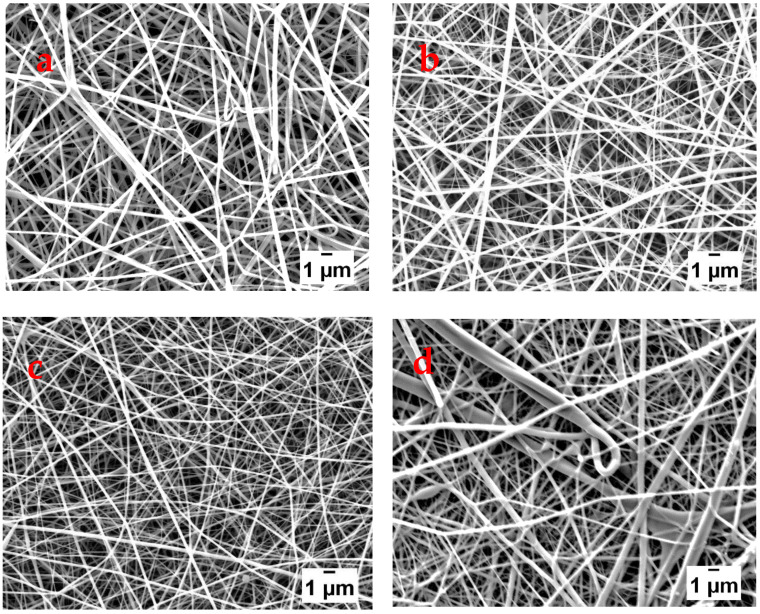
SEM images of nanofibers fabricated using applied voltages of (**a**) 10, (**b**) 13, (**c**) 15, and (**d**) 17 kV.

The SEM images of the electrospun fibers fabricated from varying applied voltages are shown in Figure 7. The images show that defect-free nanofibers were collected at applied voltages of 10 to 15 kV. When the applied voltage was increased to 17 kV the morphology of the nanofibers changed from bead free to beaded and branched nanofibers. The applied voltage also affected the diameters of the fibers, with the average fiber diameters decreasing from 146 to 112 nm with increasing applied voltage from 10 to 15kV. However, as the applied voltage was further increased from 15 to 17 kV, the average fiber diameter increased from 112 to 181 nm. This indicates that the average diameter initially decreased with an increase in the applied voltage. This effect may be associated with the increased strength of the electric field arising from the increased voltage which favored the elongation and stretching of the jet to produce finer fibers [7,20]. However, the continued increase of the applied voltage while the other parameters remained constant resulted in an even stronger electric field. Under such conditions, the rate at which the polymer solution leaves the needle tip may exceed the rate of feeding affecting the stability of the jet which may result in beaded fibers [21]. The increase in the average fiber diameter may also be an indication that the time of flight of the polymer solution from the tip of the needle to the collector was short. This might have been due to the faster acceleration of the jet towards the collector resulting in a shorter time for the stretching and elongation before reaching the collector [22].

**Figure 8:**
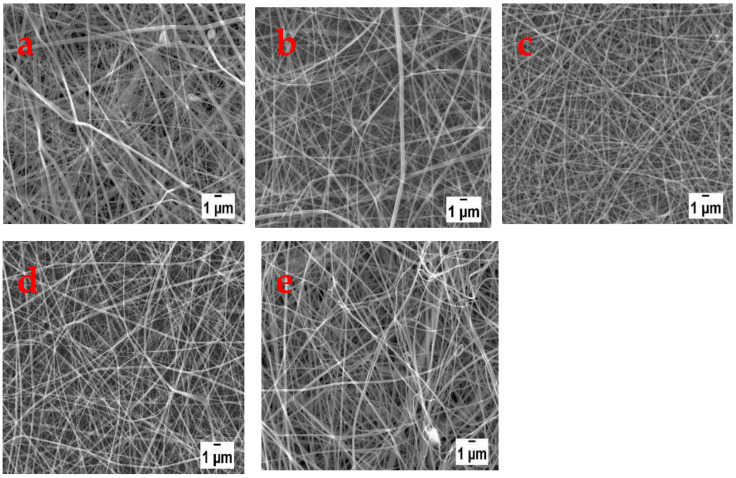
SEM images of nanofibers electrospun at tip to collector distances of (**a**) 6, (**b**) 8, (**c**) 10, (**d**) 12, and (**e**) 14 cm.

The distance from the tip of the needle to the collector affects the time of flight of the polymer jet from the tip of the needle to the collector, while at the same time also influencing the electric field strength [22]. So, the shorter the distance the faster for the polymer jet to reach the collector and the stronger the electric field under which electrospinning takes place. The effect of the tip to collector distance on electrospun nanofibers is shown in Figure 8. When the collector was placed 6 cm away, beaded morphology was observed. When the distance increased to 8 cm and above bead free nanofibers were collected. This implies that at 6 cm the solvent could not completely evaporate before the jet reached the collector due to the proximity. The average diameters of the nanofibers decreased from 141 to 119 nm as the distance increased from 6 to 14 cm. This is an indication that increasing the distance did not only provide enough time for the solvent to evaporate but also allowed the polymer jet to have enough time for elongation and thinning. However, at a distance of 14 cm, the nanofibers were frayed and rough which could be an indication that the distance was too long.

**Figure 9:**
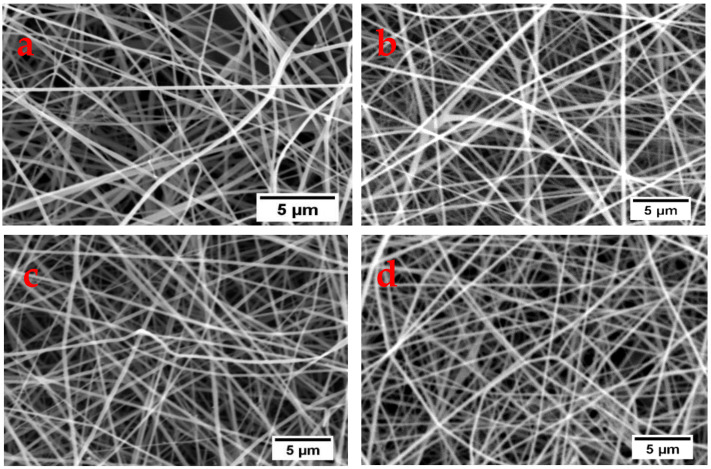
SEM images of nanofibers electrospun under optimum conditions from (**a**) CaCl_2_, (**b**) Ca(NO_3_)_2_, (**c**) LiCl, and (**d**) LiBr.

**Figure 10:**
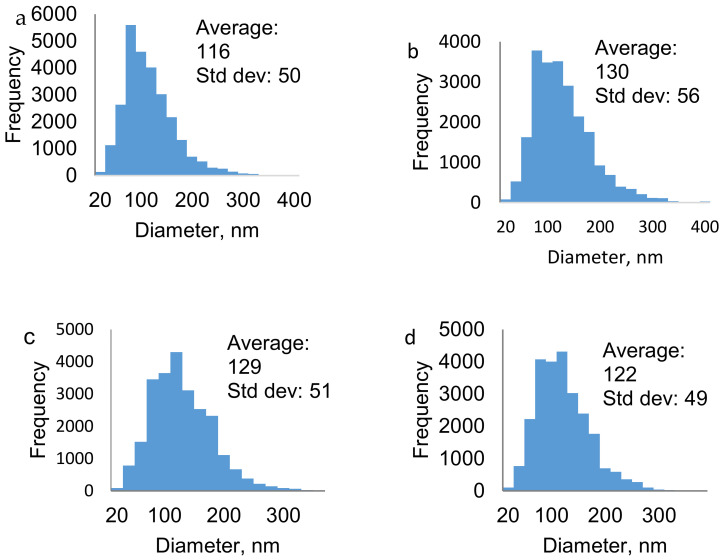
Diameter distribution of (**a**) CaCl_2_, (**b**) Ca(NO_3_)_2_, (**c**) LiCl, and (**d**) LiBr nanofibers.

The SEM images of nanofibers electrospun under optimum conditions are shown in Figure 9 and the corresponding diameter distributions for the nanofibers are shown in Figure 10. The average fiber diameters of 116, 130, 129, and 122 nm were obtained using films from CaCl_2_, Ca(NO_3_)_2_, LiCl, and LiBr, respectively. The optimum conditions were a flow rate of 6 µL min^−1^ and an applied voltage of 15 kV for all the polymer solutions of the different films. While the polymer concentration of 10 wt% was optimum for CaCl_2_, LiBr, and Ca(NO_3_)_2_ and 13 wt% for LiCl, and tip to collector distances of 12 cm for CaCl_2_ and LiBr, 10 cm for Ca(NO_3_)_2_ and 14 cm for LiCl nanofibers.

### 2.3. Thermal Properties

**Figure 11:**
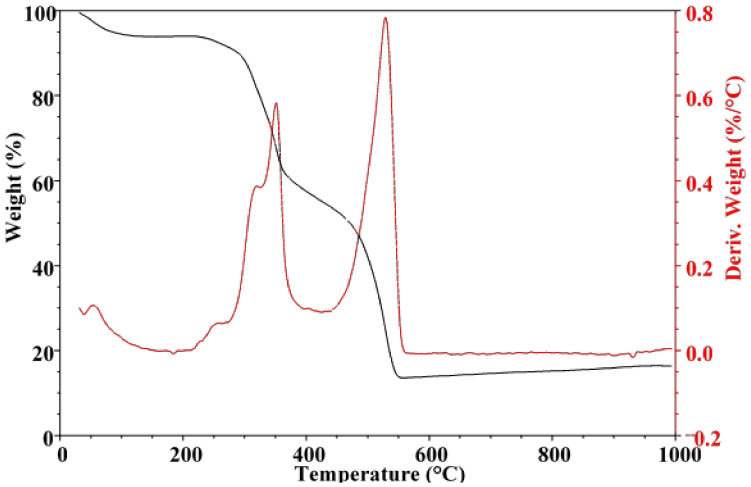
Thermogravimetric (TG) and derivative TG curves of degummed silk fibroin (SF).

The thermal properties of the degummed silk fibroin were investigated, and the thermogravimetric (TG) and corresponding derivative TG curves are given in Figure 11. An initial weight loss of about 5% which proceeds up to about 145 °C occurred generally attributed to the loss of moisture. The fibers were stable up to about 240 °C, thereafter, the silk fibroin experienced about 90% weight loss through a series of decomposition steps up to about 550 °C beyond which the fiber remained at a constant weight. A decomposition step is observed from 240 to 271 °C due to molecular motion of the silk fibroin chains because of heating resulting in the loss of low-temperature volatile compounds [23]. This was followed by two main regions of significant weight loss of the fiber. The first with a weight loss of 36% occurred from 271 to 400 °C with the derivative TG curve showing a signal maximum of around 351 °C. This step is associated with the disintegration of the intermolecular side chains and cleavage of peptide bonds [24,25]. The fiber then gradually lost less than 10% of its weight between 370 and 470 °C. About half of the fibroin had decomposed around 470 °C after about 45 min of the analysis. The second significant weight loss of about 44% occurred from 471 to 550 °C with a signal maximum of around 531 °C. This last step is has been associated with complete disintegration of the β-sheets of the main chain [24].

**Figure 12:**
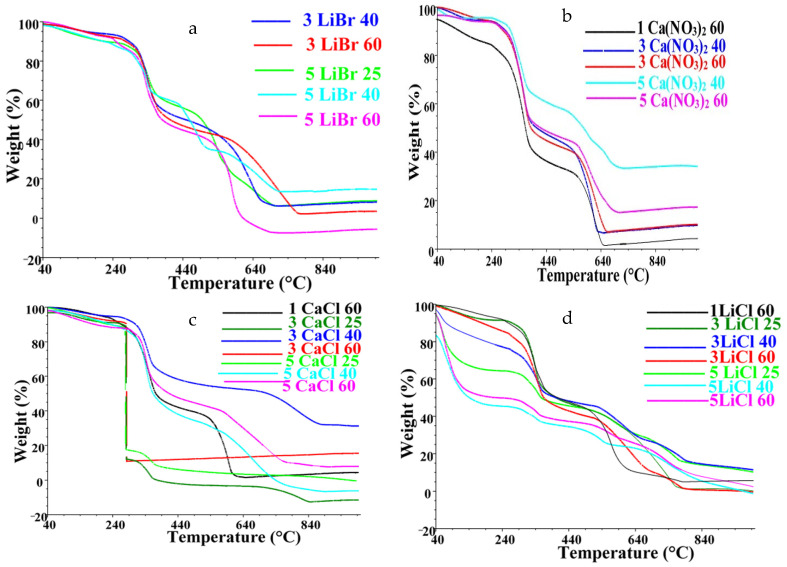
TG curves of silk fibroin films for (**a**) LiBr, (**b**) Ca(NO_3_)_2_, (**c**) CaCl_2_, and (**d**) LiCl.

The thermal patterns of the films given in Figure 12 primarily show decomposition patterns with an initial weight loss of about 5–12% corresponding to water evaporation. This was followed by a minor decomposition from about 240 °C and was immediately followed by two major decomposition steps. The first major decomposition step showed maximum degradation peaks centered between 335 and 345 °C with a corresponding weight loss of about 40%. The films experienced the final decomposition step associated with complete chain disintegration above 540 °C. Half of the fiber decomposed after about 40 min of the analysis, and this was observed at temperatures between 340 and 450 °C. The results show a decomposition pattern characteristic of silk fibroin of *A. mimosae* with minor deviations. Such deviations are an indication that processing of the fiber in some of the solvent systems only resulted in minor effects on the thermal properties.

Some films showed significant deviations from the expected thermal decomposition pattern. For instance, films cast from LiCl (Figure 12d) showed a very high-water content of up to 44%. The water content increased with an increase in salt concentration, thus, films cast from 5 wt% LiCl processed at 60 °C had the highest water content. This indicated that an increase in the LiCl concentration may have resulted in more active sites available for absorption of water molecules which negatively impacted the thermal stability of the films. The unique property might be directly related to the hygroscopicity of LiCl salt which could have resulted in traces of water molecules on the surface of the films. The high moisture content affected the thermal stability of the films which resulted in the films losing 50% of their initial weight after about 18 min of the analysis and this was observed around 212 °C. The least affected films from the LiCl solvent systems batch were those cast from 1 wt% at 60 °C with about 6% moisture content and 50% weight loss around 402 °C. Other films that did not follow the expected thermal decomposition pattern were those cast from 3 wt% CaCl_2_ systems at 25 and 60 °C (Figure 12c) which experienced a sudden sharp drop around 280 °C corresponding to about 80% weight loss. Furthermore, it took about 26 min for half of the films to decompose. This suggests that the films were less stable than those produced at 40 °C for the same salt concentration. It is suspected that the differences in thermal behavior might be associated with the influence of processing temperatures. Tretinnikov and Tamada also reported that the formation of β-sheets was favored at elevated temperatures, however, beyond a certain temperature the β-sheet content decreased [26]. Overall, the results show that some solvent systems induced only mild effects to the silk fibroin during processing, while others were harsh resulting in changes in the thermal stability of the fibroin.

**Figure 13:**
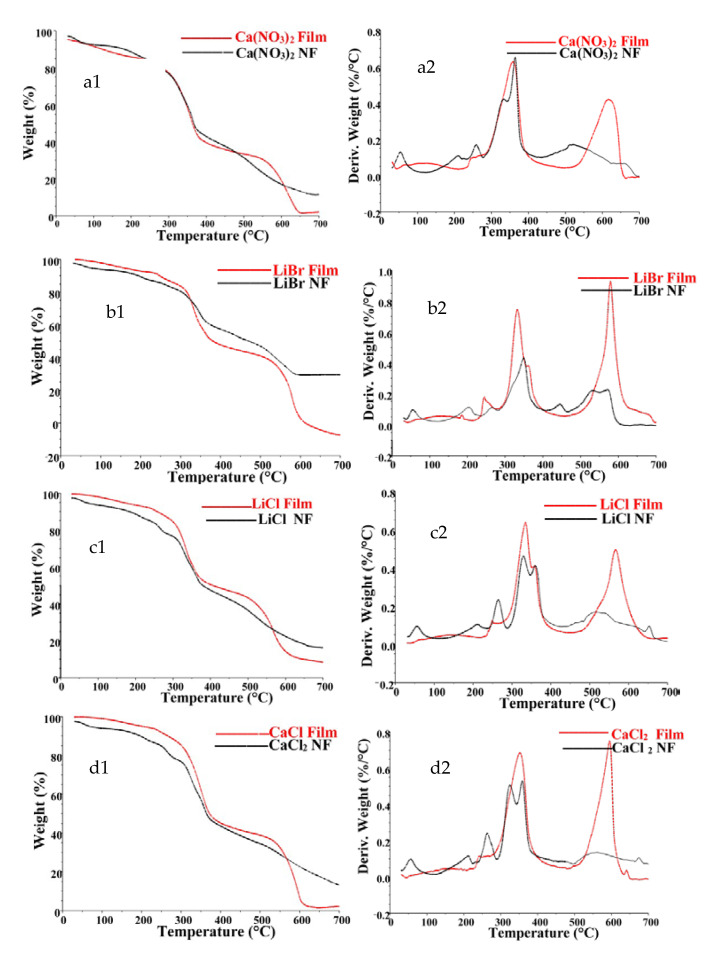
TG curves of (**a1**) Ca(NO_3_)_2_, (**b1**) LiBr, (**c1**) LiCl, and (**d1**) CaCl_2_. And corresponding derivative TG curves of (**a2**) Ca(NO_3_)_2_, (**b2**) LiBr, (**c2**) LiCl, and (**d2**) CaCl_2_ films and corresponding nanofibers.

Figure 13 shows the thermal properties of the electrospun nanofibers and the films from which the electrospinning solutions were prepared. The nanofibers also showed an initial weight loss of up to 5% corresponding to water evaporation, followed by a minor decomposition step from 220 to 280 °C. The major decomposition step occurred between 280 and 400 °C, with a peak maximum from about 330 to 370 °C. This decomposition step resulted in a weight loss of up to 37%. About 50% weight loss of the nanofibers took place after 35 min at temperatures around 365 °C. The nanofibers did not show the second major decomposition step observed for the films, suggesting low crystallinity of the nanofibers. This may have been caused by the degradation of the β-sheets of the main chain during processing [27].

### 2.4. Structural Characteristics

**Figure 14:**
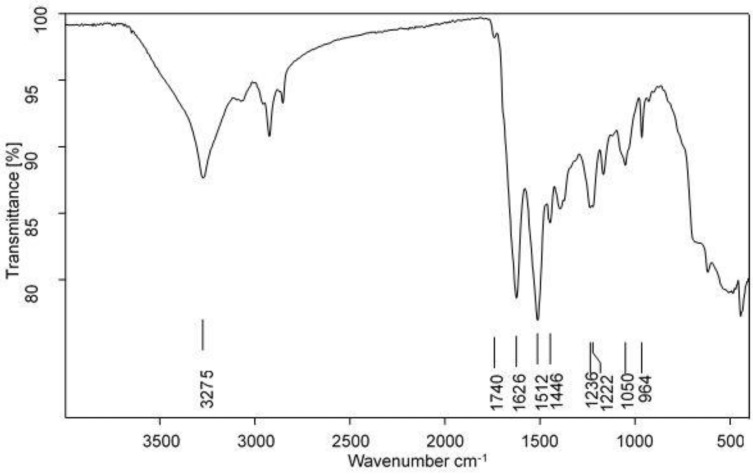
FTIR of degummed SF.

The FTIR analysis was carried out to get information on the conformation of the silk fibroin based on the position of the bands. Figure 14 shows an absorption band around 3275 cm^−1^ due to N-H stretching [28]. Other bands around 1740 cm^−1^ assigned to C=O stretching vibrations, strong amide I and amide II absorption bands around 1626 cm^−1^ (C=O stretching bands) and 1513 cm^−1^ due to N-H bending and C-H stretching, respectively, indicating β-sheet molecular conformation [10,29]. Another band also indicative of β-sheet conformation from both poly(Gly-Ala) and poly(Ala) linkages was observed around 1446 cm^−1^ [10]. Amide III twin bands were observed around 1236 cm^−1^ due to C-N stretching coupled with N-H bending and 1222 cm^−1^ due to C-H bending and C-N stretching coupled with N-H bending which are characteristic of polypeptide poly-L-alanine corresponding to a combination of random coils and β-sheets, respectively [10,30,31]. The bands around 1051 and 964 cm^−1^ have been associated with the presence of poly(Gly-Ala) and poly(Ala) sequences, respectively, in the amide V region bands around 685 cm^−1^ corresponding to β-sheet [10]. The absorption pattern of the degummed silk fibroin reveals structural conformation rich in β-sheets which is characteristic of the silk II form.

**Figure 15:**
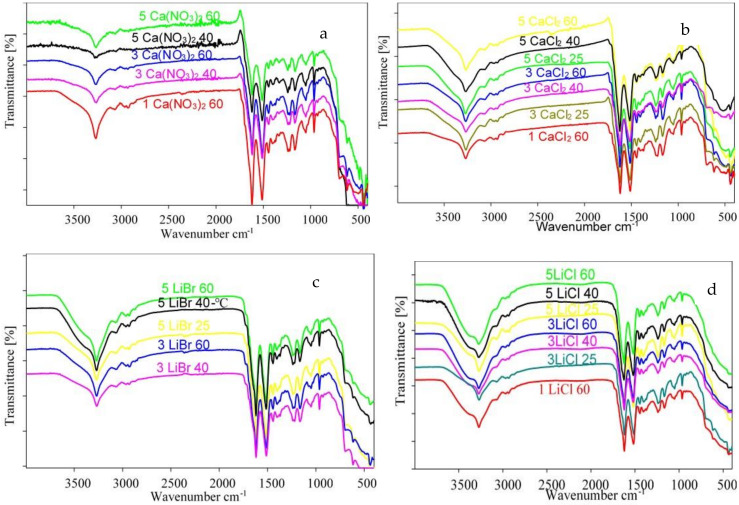
FTIR spectra for films produced by (**a**) Ca(NO_3_)_2_, (**b**) CaCl_2_, (**c**) LiBr, and (**d**) LiCl solvent systems.

The FTIR was used to investigate any changes in the secondary structure of the silk fibroin that might have taken place during processing. The FTIR results in Figure 15 shows absorption bands characteristic of the wild silk fibroin albeit some with minor shifts. This means that no major disruptions were observed for the β-sheets in the amide I (1600–1700 cm^−1^) and II (1500–1600 cm^−1^) regions as well as the poly(Gly-Ala) and poly(Ala) sequences. Some disruptions were observed in the amide III region in which the intensity of the β-sheets band around 1222 cm^−1^ was weaker while the band around 1236 cm^−1^ remained more noticeable in films. This change appeared to be more pronounced as salt concentration increased to 5 wt% regardless of salt type and temperature. The disruption was minimal in films produced with LiBr, while the same band in films produced with Ca(NO_3_)_2_ and CaCl_2_ was transformed to a shoulder. The most affected films were those produced in 5 wt% LiCl in which the β-sheets band was transformed into a very weak shoulder that almost completely disappeared. Films produced at 3 wt% CaCl_2_ and LiCl solvent systems also showed a similar conformational disruption. More disruptions on the LiCl films were observed for the β-sheets band around 695 cm^−1^ in which the band completely disappeared for resultant films from 5 wt% solvent systems. Another interesting change was observed at high wavenumbers between 3676 and 3111 cm^−1^. The resultant films cast from LiCl, CaCl_2_, and LiBr solvent systems showed band broadening particularly films produced from 5 wt% of the salts. These salts are hygroscopic and commonly used as dehumidifiers and of the three LiCl has been reported to be the most efficient [16]. Similarly, in this study, the stronger hygroscopicity of LiCl was evident as the band broadening effect was more pronounced for films produced from LiCl. As a result, a new band was observed around 3352 cm^−1^ which was attributed to O-H stretching which overlapped with the band around 3268 cm^−1^ attributed to N-H stretching [32]. This agrees with the TGA results in which high water content was observed for films cast from 5 wt% LiCl solvent systems.

The data provided by the FTIR further highlights the effect of salt type coupled with the salt concentration on the integrity of the silk structure. Based on the extent of disruptions on the properties of the degummed silk fibroin according to the FTIR and TGA results, it was possible to select films produced under mild conditions. Furthermore, the ability and extent of the solvent system to dissolve the silk fibroin was also considered when selecting mild conditions. Therefore, 1 wt% CaCl_2_, LiCl, and Ca(NO_3_)_2_ and 5 wt% LiBr all processed at 60 °C were considered to be prepared under mild conditions.

**Figure 16:**
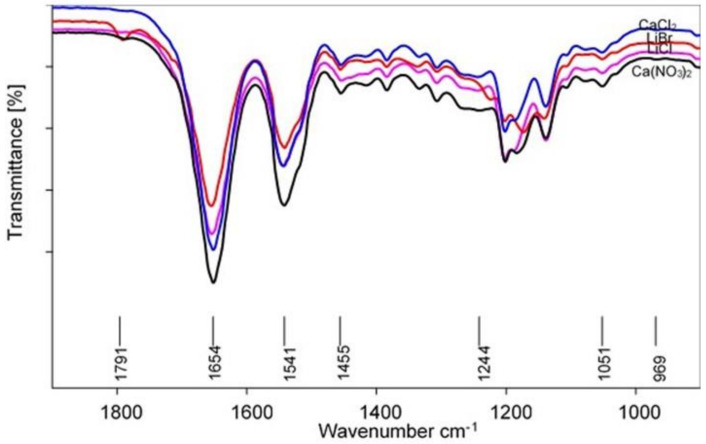
FTIR spectra for electrospun nanofibers.

Major changes in band positions were observed as shown on the FTIR spectra in Figure 16. The amide I and II regions of the nanofibers were characterized by bands indicating α-helices at 1654 and 1541 cm^−1^, respectively. The bands around 1446 and 1372 cm^−1^ shifted to 1455 and 1384 cm^−1^, respectively, which might have been due to conformational changes in the methyl group of the alanine residues [33]. Moreover, the β-sheets observed in the amide V around 693 cm^−1^ were replaced by α-helices observed around 656 cm^−1^. Nanofibers spun from films produced from LiBr and Ca(NO_3_)_2_ showed extra bands around 1791 and 1788 cm^−1^, respectively, which were not observed for nanofibers spun from CaCl_2_ and LiCl films. These bands are qualitatively similar to the band β-sheets observed around 1740 cm^−1^ for the degummed SF. Weak β-sheets were observed in the amide III region around 1223 cm^−1^ for LiBr nanofibers, while the for the other nanofibers showed a band around 1244 cm^−1^. Unlike the conformational structures of fibroin and films which were rich in β-sheets, the nanofibers were found to mainly constitute α-helices. These results indicated that further processing of silk fibroin from films to nanofibers induced conformational transition from the stable silk II structure to the less stable silk I structure.

**Figure 17:**
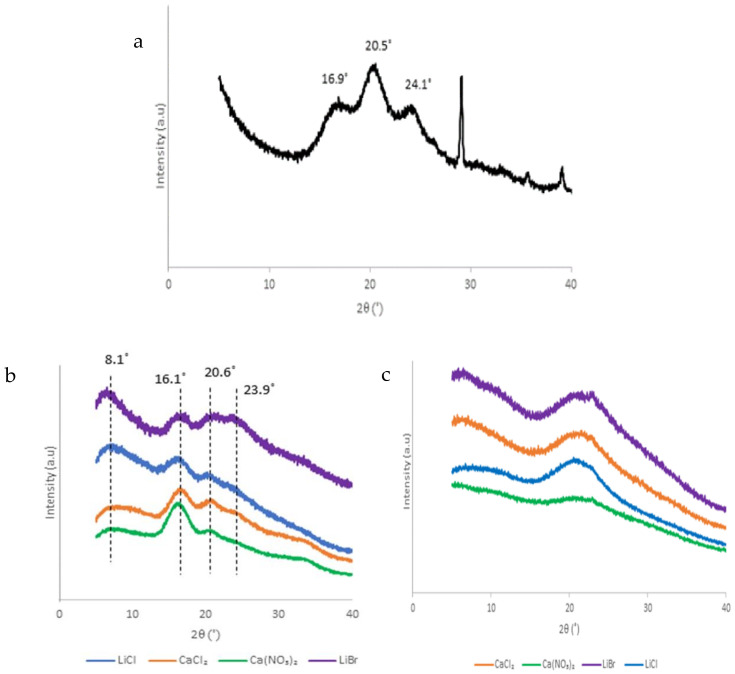
XRD diffractograms of (**a**) degummed SF, (**b**) films, and (**c**) nanofibers.

The XRD patterns (Figure 17) were used to determine the diffraction patterns to give information on the crystallinity of the different silk forms. The XRD diffractogram for silk fibroin (Figure 17a) showed diffraction peaks characteristic of β-sheet crystallinity around 16.9 and 20.5 and 24.1° corresponding to crystalline spacings of 5.2 and 4.3 Å and 3.7 Å, respectively [29]. The X-ray diffraction profiles for the films produced from the mild solvent systems given in Figure 15b show diffraction peaks around 8.1, 16.1, and 20.6° with corresponding crystalline spacings of 10.9, 5.5, and 4.3 Å, respectively. These peaks all correspond to β-sheet crystallinity [34]. An additional peak of 23.9° indicative of β-sheets with a spacing of 3.7 Å was observed only for LiBr produced films. On the other hand, the nanofibers (Figure 15c) had a broad diffraction peak centered around 21.7° typical of amorphous silk structure. This is a further confirmation processing induced disruption of the structure of the SF from the silk II to silk I form.

### 2.5. Post Treatment of Nanofibers

**Figure 18:**
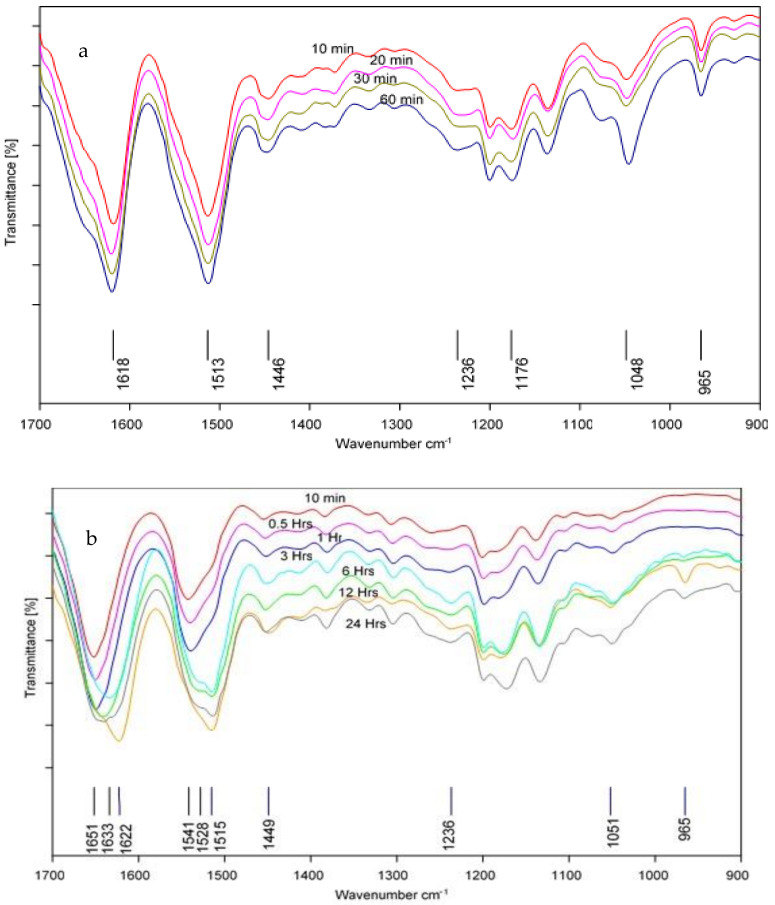
FTIR Spectra of (**a**) methanol and (**b**) water vapor annealed nanofibers.

The electrospun nanofibers were water-soluble, whereas the degummed silk fibroin and films were insoluble. This was an indication of degradation of the crystalline structure of the fibroin which resulted in the loss of mechanical strength thus the water solubility [8,27]. Post treatment to induce crystallinity using methanol and water vapor annealing were investigated and the FTIR was used to determine conformational changes. The FTIR spectra for the treatments are given in Figure 18. The methanol treated nanofibers show bands around 1618 cm^−1^ (amide I) and 1513 cm^−1^ (amide II). Moreover, the bands observed around 1455 and 1244 cm^−1^ in the untreated fibers shifted to 1446 and 1236 cm^−1^, respectively. The band shifts are evidence of conformational changes from the α-helices which characterized the untreated nanofibers to β-sheet conformation. Furthermore, the intensity of the β-sheet bands around 964 cm^−1^ reappeared for the treated nanofibers also indicating an increase in the β-sheet crystallinity. This indicates the successful conformational transition from the α-helical, silk I structure to the more stable β-sheet, silk II structure. For the water vapor treated nanofibers, the spectra, no significant structural changes took place in the first hour of the treatment with the amide I and II bands at 1651 and 1541 cm^−1^, respectively. However, as the treatment time increased to 3 h, the amide I band shifted to 1647 with a shoulder at 1638 cm^−1^, likewise, the amide II band shifted to 1528 with a shoulder around 1515 cm^−1^. After 6 h of the treatment, the shoulder at 1647 cm^−1^ disappeared and a single band was observed around 1640 cm^−1^, while the bands in the amide II region remained at almost the same position. After 12 h, the band around 1244 shifted to 1238 cm^−1^, and the poly-alanine band around 966 cm^−1^ reappeared and grew stronger for nanofibers treated for 24 h. Moreover, after 24 h, the amide I band shifted to 1622 cm^−1^ and the shoulder around 1531 cm^−1^ in the amide II region disappeared, only a single band around 1515 cm^−1^ remained. The band shifts were indicative of structural rearrangement from α-helical conformation to β-sheet conformation.

Comparatively, the results indicate that the methanol treatment was rapid and effective even within 10 min of treatment the β-sheet content had increased significantly. However, with the water vapor treatment, the transition happened over time with some conformational changes observed only after about 3 h of the treatment. Furthermore, the spectra for methanol treated nanofibers reveal high β-sheet content and this was evident also in the brittle state of the treated nanofibers. The temperature–time dependence on the effectiveness of the water vapor annealing process has been reported, in which vapor annealing at a high temperature over a shorter period had similar results with those annealing at a lower temperature over a longer time [34]. By extension, the treatment time in our study could be shortened by annealing at higher temperatures.

## 3. Materials and Methods

### 3.1. Chemicals and Reagents

Analytical grade Na_2_CO_3_ (≥99%), CaCl_2_ (≥93%), Ca(NO_3_)_2_·4H_2_O (≥99%), LiBr (≥99%), and LiCl (≥99%) (Sigma Aldrich, Steinheim, Germany). HPLC-grade methanol and formic acid (99%) (Merck, Modderfontein, South Africa) and trifluoroacetic acid (99%) (Sigma Aldrich, Steinheim, Germany).

### 3.2. Extraction of Silk Fibroin

*Argema mimosae* cocoons were collected from the Kingdom of Eswatini (former Swaziland). The cocoons were dissected to remove twigs and dirt, then thoroughly washed with deionized water. The pure silk fibroin was separated from sericin in a two-step degumming process using 0.024 mol dm^−3^ followed by 0.01 mol dm^−3^ Na_2_CO_3_ aqueous solutions. In each case, 1 g of the cocoon was boiled in 40 mL of the Na_2_CO_3_ aqueous solution for 90 min. The fibers were collected and thoroughly washed with warm deionized water and finally allowed to dry under ambient conditions. The degummed silk fibroin was then characterized using different techniques.

### 3.3. Dissolution Studies and Preparation of Films

The solvation of the degummed silk fibroin was carried in various salt–formic acid solvent systems. The influence of salt type (CaCl_2_, Ca(NO_3_)_2_, LiBr and LiCl), salt concentration (1–5 wt%) and processing temperature (25–60 °C) on the dissolution of the fibroin was determined. The general procedure involved continuous magnetic stirring at 200 rpm for 15 min to facilitate the dissolution of the fibroin. The regenerated silk fibroin solutions were centrifuged to separate the undissolved silk fibroin and the supernatant solutions were cast on polystyrene Petri dishes and kept open under a fume hood until dry. The resultant films were immersed in deionized water to wash off the residual salt and formic acid, then films were air-dried. The films were characterized using different techniques to investigate possible changes that may have occurred to the degummed silk fibroin during processing. The undissolved silk fibroin, on the other hand, was collected and thoroughly washed with deionized water to remove any residual salt and formic acid, followed by drying in an oven at 40 °C. The dry residue was used to calculate the extent of dissolution of the silk fibers using Equation (1).

(1) $$Solubility\;\left(\%\right)=\left(w_{1}-w_{2}\right)/w_{1}\times 100$$

where: $w_{1}$ is the initial weight of silk fibroin (g) and $w_{2}$ is the weight of undissolved silk fibroin (g)

### 3.4. Electrospinning

Silk fibroin films fabricated under the best conditions were dissolved in trifluoroacetic acid with constant stirring (150 rpm) at 60 °C until completely dissolved to obtain a silk fibroin polymer solution ready for electrospinning. The influence of electrospinning parameters on the morphology of the nanofibers was investigated. These included the effect of concentration of regenerated silk polymer solution (5–17 wt%), distance from the tip of the needle to the collector (6–14 cm), flow rate (4–10 μL min^−1^) and applied voltage (10–17 kV). The collected electrospun nanofibers were characterized. Finally, the nanofibers were treated using methanol (10–60 min) and water vapor (10 min to 24 h) followed by under a stream of nitrogen.

### 3.5. Characterization

The secondary structure and conformation of the different silk forms were studied using FTIR-ATR, Vertex 70 version with OPUS 7.5 software (Bruker Optik GmbH, Ettlingen, Germany). To determine the thermal properties, thermogravimetric analysis (TGA) was performed using an SDT Q600 V20.9 (TA Instruments, New Castle, DE, USA). The samples were subjected to a heating rate of 10 °C min^−1^ up to 1000 °C under nitrogen gas with a flow rate of 10 mL min^−1^. The X-ray diffraction (XRD) patterns were measured by a Rigaku Smartlab XRD (Rigaku, Neu-Isenburg, Germany) diffractometer using Cu Kα radiation (λ = 1.54 Å) in the 2θ range of 5–40°. The morphology of the silk fibroin nanofibers was observed using a JEOL-JSM-6010 Plus/LA scanning electron microscope (JEOL, Tokyo, Japan) following sputter-coating with a gold layer.

## 4. Conclusions

*A. mimosae* wild silk fibroin was successfully dissolved using solvent systems containing LiBr, Ca(NO_3_)_2_, CaCl_2_, and LiCl salts dissolved in formic acid. The choice of dissolution conditions should take into consideration factors that may affect the integrity of the silk fibroin as well as the effectiveness of the dissolution parameters used. In this study, low salt concentrations were generally preferred and gave silk fibroin forms with minimal disruption. The actual salt ions participating in the solvent systems were found to influence the dissolution process, with the Cl^−^ ions showing stronger solvation power. Following successful dissolution of the fiber in some of the solvent systems, films and nanofibers were fabricated. The resultant films were mainly characterized by β-sheets (crystalline conformation) while fabricated nanofibers were mainly characterized by less stable random coils and α-helices. Treatment of the nanofibers with methanol and water vapor effectively induced conformational changes which confirmed that β-sheets crystallinity was restored.

## Data Availability

The data presented in this study are available on request from the corresponding author.
